# Scald Fermentation Time as a Factor Determining the Nutritional and Sensory Quality of Rye Bread

**DOI:** 10.3390/foods14060979

**Published:** 2025-03-13

**Authors:** Ruta Murniece, Sanita Reidzane, Vitalijs Radenkovs, Evita Straumite, Anete Keke, Eeva-Gerda Kobrin, Dace Klava

**Affiliations:** 1 Food Institute, Latvia University of Life Sciences and Technologies, Riga Street 22, LV-3004 Jelgava, Latvia; 2 Division of Smart Technologies, Research Laboratory of Biotechnology, Latvia University of Life Sciences and Technologies, Rigas Street 22b, LV-3004 Jelgava, Latvia; 3 Institute of Horticulture (LatHort), Graudu Street 1, LV-3701 Dobele, Latvia; 4 AS TFTAK (Center of Food and Fermentation Technologies), Mäealuse 2/4B, 12618 Tallinn, Estonia

**Keywords:** rye bread, sourdough, fermentable sugars, fructans, phytic acid

## Abstract

This study investigates the effect of extended rye scald fermentation times (12–48 h) on its biochemical properties and rye bread’s nutritional and sensory qualities. Traditional rye bread production in Latvia involves prolonged fermentation with lactic acid bacteria (LAB), a process that influences the bread’s acidity, sugar content, and concentrations of organic acids, fructans, and phytates. Scald fermentation was analyzed at intervals of 0, 12, 24, 36, and 48 h to monitor microbial activity, particularly LAB population dynamics. Organic acids and sugar profiles were analyzed using HPLC, while phytic acid and fructan concentrations were determined using the Phytic Acid Assay Kit (K-PHYT) and Fructan Assay Kit (K-FRUC). Sensory evaluation assessed attributes including aroma, sour and sweet taste, stickiness, and floury aftertaste. A rapid pH decrease and increased total titratable acidity (TTA) after 12 h confirmed scald’s suitability as a substrate for *Lactobacillus delbrueckii* metabolism. Lactic acid content increased 13.8-fold after 48 h. Combined scald and dough sourdough fermentation reduced phytic acid by 20% and fructans by 49%, improving mineral bioavailability. Extending fermentation beyond 24 h showed no significant differences in physicochemical parameters, although it improved sensory quality, reduced stickiness, balanced sweet–sour flavors, enhanced aroma, and minimized floury aftertaste.

## 1. Introduction

Rye flour, obtained from the grain of *Secale cereale* L., is an important food crop in the northern regions of Europe, valued for its nutritional benefits [1] and distinctive flavor profile, particularly in the production of rye bread [2]. However, rye presents significant challenges in bread making compared to wheat flour due to its chemical composition, higher water absorption capacity, and unique amylase enzyme activity. To ensure proper structure and enhance flavor, sourdough fermentation is commonly employed in the production of rye bread. Rye bread is of considerable interest to consumers due to its higher levels of essential amino acids (lysine), dietary fibers, essential minerals, folates, tocopherols, tocotrienols, lignans, phytosterols, and phenolic acids, all of which are significant for human health [2,3]. Furthermore, rye breads have been associated with positive health effects, including appetite suppression and a reduced risk of colon cancer and hypercholesterolemia [4].

One of the traditional methods in northern countries for rye bread production involves a multi-stage process, including scald preparation, fermentation, and dough preparation and fermentation [5]. Rye flour contains amylases that degrade starch into fermentable sugars, such as glucose and maltose, during scald saccharification, a process further enhanced by diastatic malt [6]. This starch degradation influences the initial sugar profile of rye dough and provides essential substrates for microbial fermentation [7]. Lactic acid bacteria (LAB) metabolize these sugars, reducing residual sugars in the bread product [8]. This consumption impacts the sweetness of scald and bread and influences overall flavor development. Scalding improves the dough’s rheological properties and breadcrumb hardness during storage [9]. Rye scald can be sourdough fermented with a combination of lactic acid bacteria and yeast. Since scalding maintains the mixture at a relatively high temperature (approximately 50 °C), fermentation can be performed using adapted thermophilic *Lactobacillus delbrueckii* bacteria [10], which are characterized by severe lactic acid production and have been found in liquid (Type II) sourdough [11]. Extended fermentation times significantly affect microbial populations within sourdough systems [12]. The total fermentation time for bread production from scalded rye flour varies between 6 and 48 h, more often 24 h [5]. LAB species and fermentation time influence the bread’s texture, flavor, and nutritional value, including acidity, sugar, and organic acid content.

Previous studies by Pontono et al. [13] on LAB led sourdough fermentation to produce a range of organic acids, such as lactic acid and acetic acid, which enrich the sensory attributes of rye bread. Producing organic acids during sourdough fermentation is vital in enhancing flavor and promoting the health benefits of rye bread consumption. Lactic acid contributes a tangy taste, while acetic acid adds complexity and depth [14]. However, the formation of organic acids is influenced by several factors, including the specific LAB strains and environmental conditions such as temperature and fermentation duration [15]. Organic acids produced during sourdough fermentation improve the flavor characteristics of the rye bread, promote digestibility, and enhance nutrient bioavailability [16,17].

Fermentation processes that increase organic acids may also reduce the content of antinutritional factors like phytic acid, making nutrients in the bread more bioavailable. Studies have shown that rye sourdough bread has improved levels of essential minerals compared to non-fermented rye bread. The acidic environment LAB creates during fermentation helps solubilize minerals, making them more accessible for absorption in the gastrointestinal tract [18].

Rye grains contain 0.54–1.46% phytates (myo-inositol 1,2,3,4,5,6-hexaphosphate), which form insoluble complexes with divalent minerals like zinc (Zn), iron (Fe), and calcium (Ca), reducing their [19,20,21]. Phytates, concentrated in the outer layers of grains, are a significant antinutrient in cereal-based foods [22]. Although the degradation of phytates in rye bread production has been underexplored, studies show that extended fermentation times can reduce phytic acid to lower inositol phosphates and free phosphate [23]. Rye contains 1.82 mg g^−1^ dry weight (dw) of phytic acid [24], and enzymatic breakdown of phytic acid occurs during fermentation, particularly with lactic acid bacteria (LAB) and yeasts. Phytase, the enzyme responsible for hydrolyzing phytates, is present in indigenous rye phytases and microbial cultures in sourdough [25,26]. LAB enhances mineral bioavailability by increasing phytase activity during extended fermentation (approximately 12 h), and sourdough-induced acidification to a pH of 5.5 further promotes phytate breakdown [27].

Rye bread is considered one of the richest sources of fructans, a type of fructose polymer, with fructan content ranging from 4.6 to 6.6 g 100 g^−1^ dw [28]. Fructans and their fermentation products are valued for their prebiotic properties and health benefits, including promoting digestion, intestinal barrier function, and resistance to intestinal infections [29,30]. However, fructans can also trigger symptoms in individuals with gastrointestinal disorders such as irritable bowel syndrome, which affects a significant portion of the global population [31,32]. Extended fermentation (12–48 h) can reduce fructan content by up to 69%, improving rye bread digestibility by breaking down complex carbohydrates and lowering the level of fermentable oligosaccharides, disaccharides, monosaccharides, and polyols (FODMAPs), benefiting individuals with fructose malabsorption or IBS [19,20,23]. Specific LAB strains and optimized conditions are often required for significant degradation [32,33,34]. The degradation of fructans during fermentation is influenced by the enzymatic activity of microbial β-fructofuranosidase, which hydrolyzes fructans into fermentable sugars such as fructose and glucose [30,34]. However, the primary degradation of fructans occurs as a result of the activity of extracellular fructanases, which are mainly produced by yeasts [30]. By optimizing fermentation conditions, it is possible to produce rye bread with reduced fructan content, making it more digestible for individuals sensitive to fructose-derived oligo- and polysaccharides [32,35].

This study investigates the effect of long fermentation (12–48 h) on the biochemical characteristics of rye scald and its influence on the nutritional and sensory properties of rye bread. The focus is to analyze changes in sugars, organic acids, and antinutrients such as phytates and fructans during extended rye scald fermentation and the influence on the bread’s composition, flavor, and texture. The objective was to determine how prolonged rye scald fermentation time influences these parameters, whether it has a significant effect, and whether it improves the functional and sensory properties of the bread. This research contributes to optimizing rye scald fermentation time to enhance the nutrition and flavor of rye bread.

## 2. Materials and Methods

### 2.1. Materials

The ingredients used in the study included rye wholemeal flour from farm Kelmeni, Ranka parish, Latvia (moisture 14.65%, falling number 262 s), and rye diastatic malt from LTD “LATMALT”, Jelgava parish, Latvia (moisture 5.39%, diastatic power 362 °WK). Additional ingredients included drinking water and salt. The sourdough for scald (Sourdough Scald—source of a LAB) and sourdough for dough (Sourdough Dough—used as a leavening agent) for fermentation were obtained from bakery Kelmeni, Ranka parish, Latvia. The functionality and application of Sourdough Scald and Sourdough Dough have been described in a previous study [9]. The parameters of the sourdoughs are summarized in Table 1.

### 2.2. Preparation and Fermentation of Rye Scald Samples

For the bread samples’ preparation, five scalds fermented for different durations were used: 0 h (unfermented), 12 h, 24 h, 36 h, and 48 h. The scalds were prepared according to the recipe (Table 2), using rye wholegrain flour combined with rye diastatic malt. The flour was mixed with hot water (92 ± 2 °C) in a dough mixer (SP45, WP Kemper, Germany, 1 speed 120 rpm) for 30 min under the lid, ensuring a stable temperature.

**Figure 1:**
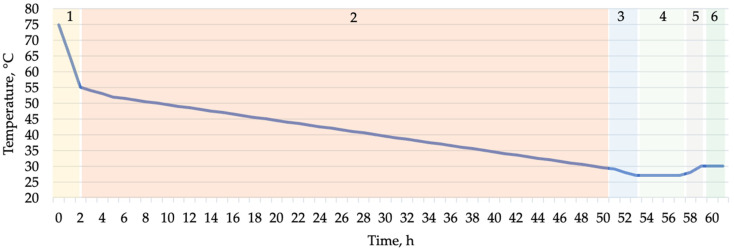
Internal scald and dough temperature changes during scald fermentation (48 h) and dough preparation. 1—scalding and saccharification (2 h), 2—fermentation with Sourdough Scald (48 h), 3—cooling (3 h), 4—scald fermentation with Sourdough Dough (4 h), 5—dough mixing (1 h), 6—deviation, shaping, and final fermentation (2 h).

The mixture was allowed to undergo saccharification for 90 min, during which the temperature of the scald decreased from 75 ± 2 °C to 55 ± 2 °C (Figure 1). Following saccharification, Sourdough Scald was added to the scald, and the mixture was fermented in wooden barrels at 30 ± 2 °C and 75% relative humidity for varying durations of 12 h, 24 h, 36 h, or 48 h. For control, an unfermented scald (0 h) was used. A separate scald was prepared for each sample.

**Figure 2:**
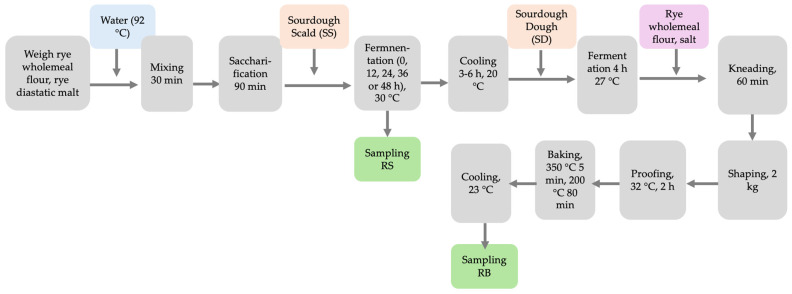
Flowchart of scald preparation, sampling, dough and bread preparation, and bread sampling.

The process flowchart is shown in Figure 2. Scald samples were collected after the fermentation stage for immediate analyses (pH, TTA). For all other analyses, the samples were frozen in a freezer (−18 °C) and subsequently lyophilized. The entire fermented scald was used for dough preparation.

### 2.3. Dough Preparation and Bread Baking

Following fermentation, the scald was cooled for 3–6 h at 20 ± 1 °C, reaching an internal temperature of 27 °C. Subsequently, Sourdough Dough was added and fermented for 4 h at 27 ± 1 °C. After fermentation, rye wholegrain flour and salt were added, and the dough was slowly kneaded for 1 h. Loaves weighing 2 kg each were shaped and proofed in a fermentation chamber (Sveba Dahlen AB, Fristad, Sweden) at 98% relative humidity and 32 ± 1 °C for 2 h. Baking was performed in oven T2 Polis (Zanolli s.r.l., Verona, Italy) at 350 ± 1 °C for 5 min, followed by 200 ± 2 °C for 80 min, until the loaf’s internal temperature reached 96 ± 1 °C. After baking, the bread was cooled on shelves (23 ± 2 °C and RH 55%) to an internal temperature of 25 ± 1 °C and then sliced. Samples were collected to immediately analyze pH and total titratable acidity (TTA). For sensory evaluation, samples were prepared from fresh bread 48 h post-baking. For further analyses, including evaluating organic acids, sugars, fructans, and phytic acid, the samples were frozen in a freezer (−18 °C) and subsequently lyophilized.

### 2.4. Lyophilization of Scald and Bread Samples

As previously described [6], the frozen scald and bread samples were lyophilized, with some modifications. Samples were placed in a FrostX 10 freeze-drier (FrostX, Gliwice, Poland) and frozen at temperatures ranging from −35 °C to −45 °C. They were then subjected to freeze-drying under vacuum conditions of 70–85 Pa, followed by freeze-free-drying at 30 °C. The total drying process took approximately 9 h. After the freeze-drying process, the samples were finely ground using a Foss Knifetec 295 Mill (Foss Analytical Co., Ltd., Suzhou, China) laboratory grinder.

### 2.5. LAB and Yeast Identification in Sourdough Scald and Fermented Scald

Microorganism identification was carried out similarly to how previously described [36]. Cells of microorganisms were collected from a lyophilized sample by differential centrifugation. The Quick-DNA Fungal/Bacterial Miniprep Kit (LOT 211352, Zymo Research Corp, Irvine, CA, USA) was used for DNA extraction. The DNA concentration from samples was measured by QubitTM 3.0 fluorometer (Thermo Fisher Scientific, Eugene, OR, USA) using QubitTM dsDNA BR Assay Kit (LOT 2806866, Thermo Fisher Scientific, Eugene, OR, USA).

The gDNA sample was utilized for sequencing library preparation, using the V4 (F515/R806) primer pair for 16S metabarcoding [37] to identify bacteria. For 16S libraries, sequencing was performed using an iSeq100 (Illumina) system, an iSeq Reagent kit v2 by 2 × 150 bp (Illumina, Queenstown, Singapore), and a dual index setup.

### 2.6. Lactic Acid Bacteria and Yeast Enumeration in Scald

Enumeration of lactic acid bacteria and yeasts in sourdough and scald samples was conducted using the plate count method described by Fabio Minervini [38]. A 10 g scald sample was mixed with 90 mL sterile salt peptone water and homogenized with a Bag Mixer (Interscience, Bois des Arpents, France). The resulting suspensions were appropriately diluted and inoculated on an MRS agar (Scharlab S.L., Barcelona, Spain). The petri plates were incubated for 72 h at 37 ± 1 °C in a jar using oxygen absorbent BD GasPak (Benex Ltd., Dublin, Ireland). The yeast incubation was performed using malt extract agar (Scharlab S.L., Barcelona, Spain). The plates were incubated for 48 h at 26 °C. Colony counts were performed using the automatic colony counter Scan 500 (Interscience, Bois des Arpents, France). Cell density was calculated as average numbers of three replicates [38] and represented as decimal logarithms of colony-forming units (CFUs) per gram of sample.

### 2.7. Determination of Physicochemical Parameters of Scald and Bread

The total titratable acidity (TTA) of scald and rye bread was analyzed according to the AACC 02-31.01 method [39]. The pH was measured using a pH meter using the AACC 02-52 [40] method (Mettler Toledo, Giessen, Germany). All samples were analyzed in triplicate.

### 2.8. Determination of Sugar Profile in Scald and Bread Samples

Lyophilized scald and bread samples were used to determine the sugar profile. The sugars (xylose, arabinose, glucose, sucrose, maltose, and fructose) were quantitatively analyzed using high-performance liquid chromatography with refractive index detection (HPLC-RID). The analysis was performed in duplicate. The methodology is described in detail in the study by Murniece et al. [6].

### 2.9. Determination of Organic Acids in Scald and Bread Samples

Organic acids (oxalic acid, tartaric acid, quinic acid, malic acid, ascorbic acid, citric acid, fumaric acid, succinic acid, lactic acid, acetic acid, and propionic acid) were quantitatively analyzed using a Shimadzu LC-20 Prominence high-performance liquid chromatograph (Shimadzu Corporation, Kyoto, Japan) equipped with a LC-20A solvent delivery system, CBM-20A system controller, DGU-20A5R degassing unit, CTO-20AC column oven, SPD-M20A PDA detector, and SIL-20AC autosampler. The organic acids were determined using the method previously described by Radenkovs et al. [41], with some modifications in sample preparation.

Samples were prepared by weighing 0.5 g of lyophilized scald or bread sample into 15 mL conical plastic tubes (Sarstedt AG & Co. KG, Numbrecht, Germany) and adding 10 mL of distilled water. The mixture was vortex-mixed vigorously for 15 min using a VORTEX 3 mixer (IKA^®^, Staufen, Germany). Then, the prepared samples were centrifuged for 10 min at 10,000× *g* and 19 ± 1 °C using a Centrifuge Pro-Research (Centurion Scientific Ltd., Stoughton, UK) to facilitate the separation of the fractions.

Before chromatographic analysis, the obtained supernatants were filtered through a 0.45 µm hydrophilic PTFE membrane filter (Macherey-Nagel GmbH & Co. KG, Dueren, Germany). The organic acids were separated using a YMC C18 analytical column (4.6 mm × 250 mm I. D., 5 µm particle size). The mobile phase consisted of a 0.05 M potassium dihydrogen phosphate buffer (KH_2_PO_4_; Sigma-Aldrich Chemie GmbH, Steinheim, Germany) with pH of 2.8, adjusted with 80–90% phosphoric acid (H_3_PO_4_; HPLC grade, Sigma-Aldrich Chemie GmbH, Steinheim, Germany) and 10 mL of acetonitrile (HPLC grade, CHROMASOLV^®^, from Honeywell Riedel-de Haën GmbH, Seelze, Germany), and the solution was diluted to the final volume of 1 L in a volumetric flask using distilled water. The HPLC analysis was carried out under isocratic conditions with a 1.0 mL/min flow rate, and the injection volume was 10 µL. The column temperature was set to 40 ± 1 °C. Detection was performed at a wavelength of 210 nm using the PDA detector. The total analysis time was 15 min. Samples were analyzed in duplicate (*n* = 2, each with 3 measurements).

### 2.10. Quantification of Phytic Acid

Phytic acid was determined using the Megazyme (Megazyme Ltd., Wicklow, Ireland) Phytic Acid Assay Kit (K-PHYT) according to the manufacturer’s protocol [42]. Measurements were performed in triplicate following the manufacturer’s instructions, utilizing the BioTek Synergy H1 Multimode Reader (Agilent Technologies, Winooski, VT, USA) at 410 nm.

### 2.11. Quantification of Fructans

Fructans were measured using Megazyme (Megazyme Ltd., Wicklow, Ireland) Fructan Assay Kit (K-FRUC) (AACC Method 32-32.01 [43]) according to the manufacturer’s protocol. The measurements were conducted in triplicate according to the manufacturer’s instructions, utilizing Helios Gamma Spectrophotometer 9423 UVG 1202E (Thermo Electron Corpora-tion, Waltham, MA, USA) at 655 nm.

### 2.12. Sensory Evaluation of Bread

A total of 47 panelists, comprising students and staff from the Food Institute, participated in the sensory evaluation after receiving basic sensory training. All panelists were informed of the study objectives, provided informed consent, and participated voluntarily. Their confidentiality was maintained, and they could withdraw at any time. Only individuals in good health, without wheat allergies or intolerances, were included in the evaluation. The rye bread sensory study was approved by the LBTU Food Institute Ethical Committee Act No. 2025/1.

The evaluation was conducted in a laboratory at the Food Institute, Latvia University of Life Sciences and Technologies, adhering to the ISO 8589:2007 [44] standard. Each panelist was presented with bread samples coded with three-digit numbers in randomized order in a single session. Water was provided as a palate cleanser.

Sensory attributes, including aroma, sour taste, sweet taste, stickiness, and floury aftertaste, were assessed using a unipolar 12 cm line scale according to ISO 4121 [45]. The scale ranged from 0 to 12, with the following criteria: aroma (0 = none, 12 = very pronounced), sour taste (0 = not sour, 12 = very sour), sweet taste (0 = not sweet, 12 = very sweet), stickiness (0 = not sticky, 12 = very sticky, feels like it is not baked), and floury aftertaste (0 = none, 12 = very pronounced floury aftertaste). Data were collected and processed using FIZZ Acquisition 2.51 software (Biosystems, Couternon, France).

### 2.13. Statistical Analysis

The collected data were compiled and initially processed using Microsoft Excel, where mean values and standard deviations were calculated to summarize the results. Statistical analyses were performed using RStudio v. 4.4.0. A one-way analysis of variance (ANOVA) was conducted to evaluate differences between groups. To identify specific group differences, post hoc comparisons were performed using Tukey’s Honest Significant Difference (HSD) test. The readxl and multcompView packages were used for data import and post hoc analysis, respectively. Statistical significance was set at (*p* ≤ 0.05).

## 3. Results

### 3.1. Effect of Fermentation Time on Scald and Bread Properties

#### 3.1.1. Changes in Microbial and Physicochemical Characteristics of Scald During Fermentation

The microbiota of fermented scald influence pH and TTA changes, affecting the bread’s nutritional and sensory properties. Therefore, the LAB and yeasts of the sourdough used for scald fermentation were identified. In the analyzed rye Sourdough Scald, which was incorporated to facilitate fermentation and is sustained through daily renewal processes, 99.8% of the obtained reads were identified as specific to the *Lactobacillus delbrueckii*. A similar result was observed when analyzing the scald microbiota after 24 and 48 h fermentation. This lactic acid bacteria have been observed in the microbiota of spontaneously fermented rye and wheat sourdoughs, as documented in previous studies [46,47] and an earlier investigation focused on fermented rye scald [9].

The enumeration of yeasts did not reveal any colonies during the fermentation of scald. Scalding reduces the number of microorganisms (LAB, yeasts, and molds) [48]. Moreover, high acidity, elevated temperatures, and prolonged fermentation time are unfavorable for yeasts’ growth.

**Figure 3:**
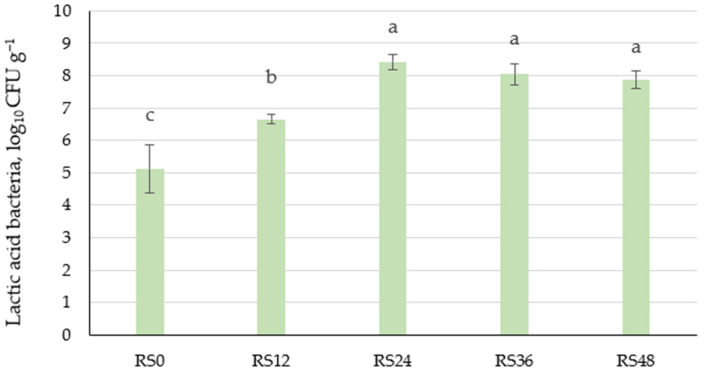
Variations in lactic acid bacteria log_10_ CFU g^−1^ in unfermented (RS0) and fermented scald samples during 48 h of fermentation (RS12—12 h, RS24—24 h, RS36—36 h, and RS48—48 h fermented). a–c indicate significant differences between the samples (*p* ≤ 0.05).

A significant (*p* ≤ 0.05) increase in LAB was noted during scald fermentation (Figure 3). LAB considerably increased during the first 12 h of fermentation from 5.1 to 6.7 log_10_ CFU g^−1^. No significant changes were observed during fermentation from 24 to 48 h, with a peak value of 8.4 log_10_ CFU g^−1^ reached at 24 h.

**Figure 4:**
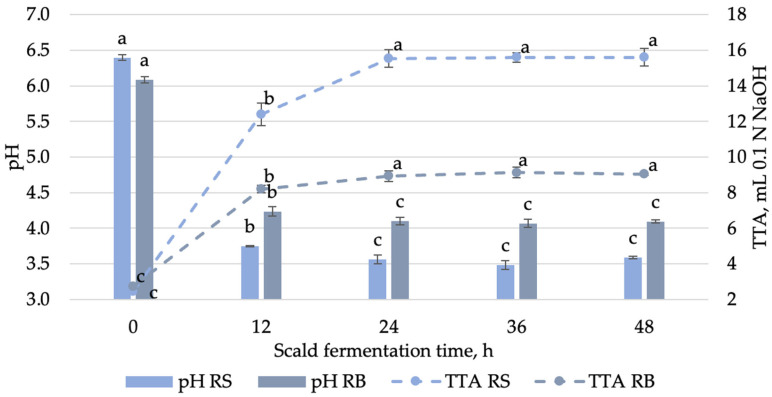
pH and TTA (mL 0.1 N NaOH) of rye scald (RS) fermented for different durations and of rye bread (RB) prepared with scald fermented for varying durations. Different letters (a–c) above the bars/points within each measurement set (TTA RS, TTA RB, pH RS, and pH RB) indicate significant differences (*p* ≤ 0.05), analyzed separately for each parameter separate for scald and bread.

During the saccharification phase of scalding, sugars (fructose, glucose, and maltose) are formed, which are necessary for the growth of the LAB [7]. *Lactobacillus delbrueckii* group bacteria show growth in high-temperature environments, 40–55 °C, as confirmed by previous studies on fermented scald [9]. The combination of optimal temperature and adequate sugar concentrations facilitated rapid LAB proliferation, as evidenced by the notable decrease in pH and increase in TTA (Figure 4).

The pH level experienced a decline from 6.40 ± 0.04 to 3.59 ± 0.02 over a 48 h fermentation period, with the most substantial reduction observed within the initial 12 h. After 24 h of fermentation, the pH and TTA remained stable, with no significant differences between samples, and were 3.5 and 15.6 mL NaOH, respectively. The final pH, typically ranging from 3.5 to 4.3, is commonly regarded as an indicator of a well-developed sourdough fermentation. Differing from our findings, Klupsaite et al. [7] reported that in scald fermentation with *Lactiplantibacillus paracasei*, the pH and TTA were 4.57 after 24 h of sourdough fermentation. This confirms the high acid production of *Lactobacillus delbrueckii*. Similar results were observed by Cizeikiene et al. [10], who studied the fermentation of wholegrain wheat sourdough at 40 °C with *Lactobacillus delbrueckii* ssp. *bulgaricus*. Their findings demonstrated a pH drop below 3.5, accompanied by a higher TTA value of 16 mL NaOH. Similarly, the pH rapidly decreased within the first 24 h, followed by stabilization over the remaining 72 h of sourdough fermentation.

The pH and TTA of bread samples prepared with unfermented scald and scalds fermented for 12, 24, 36, and 48 h followed a similar trend to that observed in the scalds at the corresponding fermentation times (Figure 4).

The bread samples consistently exhibited higher pH values (4.07 ± 0.06 to 6.09 ± 0.04) and lower TTA levels (2.7 ± 0.12 to 9.1 ± 0.31), reflecting reduced acidity compared to the scalds. These differences can be attributed to the combined effects of dough fermentation, scald acidity, and the addition of flour during bread preparation. Notably, the pH and TTA of bread samples fermented for 24, 36, and 48 h showed no significant differences, indicating relative consistency in acidity levels across these fermentation durations.

The variation in acidity levels within the fermented scald and bread, attributed to the organic acids produced by lactic acid bacteria, establishes the acid profile, which plays a critical role in shaping the bread’s attributes.

#### 3.1.2. Effect of Fermentation Time on Organic Acid Profile in Rye Scald and Rye Bread

Organic acids are mainly generated during the fermentation process. However, wholemeal rye flour inherently contains a range of acids that shape the bread’s organic acid profile. Wholemeal rye flour contained small amounts of organic acids, primarily quinic acid 0.64 ± 0.05 g 100 g^−1^ dw and malic acid 0.16 ± 0.06 g 100 g^−1^ dw, along with minor amounts of lactic, acetic, oxalic, and succinic acid. In ground rye grains, succinic acid has been found in a higher relative concentration but fumaric and malic acid in a lower concentration [49]. Cardoso et al. [50] indicated that oxalic acid, malic acid, and fumaric acid are found in trace amounts in rye bran. In contrast, citric acid (0.471 g 100 g^−1^ dw) was identified as the main acid contained in rye bran.

The organic acid profile of the fermented scald was primarily composed of lactic acid, which exhibited a rapid tenfold increase within 12 h, reaching its peak at 24 h. Beyond this point, lactic acid levels remained relatively stable, showing no significant changes after 48 h of fermentation. Other acids were present in smaller amounts (Table 3). Quinic and tartaric acid levels remained unchanged throughout fermentation, while malic and butyric acid content significantly decreased after 12 h. Lactic acid production during fermentation explains the decrease in pH and the increase of TTA in the fermented scald.

A predominance of lactic acid in the range 0.81 ± 0.06 to 2.06 ± 0.12 g 100 g^−1^ dw was observed in the bread, likely due to the addition of fermented scald to the dough, which was rich in lactic acid (Table 4). In contrast, the bread has a lower lactic acid content because a new portion of flour was added to the dough and fermented for a shorter time. Acetic, propionic, and butyric acid content showed no significant variation across all bread samples. However, their levels were substantially higher than those in scald samples. This increase was attributed to the formation of metabolites during the sourdough fermentation of the dough. Plessas et al. [51] found a lower amount of acids in bread fermented with *L. delbrueckii* ssp. *Bulgaricus* contained 0.25 g kg^−1^ of acetic acid and 2.88 g kg^−1^ of lactic acid. The fermentation time of the scald added to the dough does not affect the acetic, propionic, butyric, oxalic, quinic, and tartaric acid content.

During fermentation, *Lactobacillus* spp. metabolizes carbohydrates to produce a range of low molecular weight organic compounds, including acetic, propionic, and butyric acids. These compounds, known as short-chain fatty acids (SCFAs), play a vital role in enhancing the flavor and acidity of fermented bread [52]. Consumers of fermented wholemeal rye bread receive ready-made postbiotics, potentially enhancing their levels in the body. A study by Ros et al. [53] found that after 14 days of consuming wheat sourdough bread, the levels of SCFAs and their derivatives in feces increased, suggesting that sourdough bread consumption can enhance SCFA synthesis in the human colon.

#### 3.1.3. Effect of Fermentation Time on Rye Scald and Rye Bread Sugar Profile

Following the processes of scalding and saccharification, the fructose content in the unfermented scald was determined at 0.40 ± 0.01 g 100 g^−1^ dw (Table 5). During the fermentation phase, no fructose content was detected in the scald. The reduction in fructose can be explained by the fact that homofermentative LAB (*L. delbrueckii*) consumes it as a carbon source during fermentation [30]. The glucose amount decreased from 0.99 ± 0.05 g 100 g^−1^ dw at the initial time point (0 h) to 0.72 ± 0.08 g 100 g^−1^ dw at 12 h, after reaching a peak of 1.08 ± 0.01 g 100 g^−1^ dw at 48 h. The sucrose amount was highest immediately following the scald saccharification period, at 1.01 ± 0.09 g 100 g^−1^ dw, and it significantly decreased during fermentation, dropping to 0.25 ± 0.01 g 100 g^−1^ dw after 48 h. Sucrose can be consumed by LAB during the activity of extracellular glucansucrases or fructansucrases, leading to the formation of indigestible poly- and oligosaccharides (glucans, isomalto-oligosaccharides, fructose, levan or inulin, fructo-oligosaccharides, and glucose). *L. delbrueckii* has been identified as a positive producer of these enzymes [30]. Conversely, the maltose amount remained relatively stable throughout the fermentation process, with a modest increase from 25.33 ± 0.62 g 100 g^−1^ dw at 0 h to 27.56 ± 0.12 g 100 g^−1^ dw at 12 h. Upon evaluating the concentration of total sugars at the initial time point of 0 h, the baseline total sugar content was determined to be 30.78 ± 0.09 g 100 g^−1^ dw. At the 12 h interval, a significant increase in total sugar content was observed, measuring at 33.25 ± 0.63 g 100 g^−1^ dw, corresponding to an approximate increase of 4.8% from the baseline. No significant changes were observed for samples with a longer fermentation time (24 h, 36 h, and 48 h).

Overall, changes in sugar levels during prolonged fermentation were minor, with only slight fluctuations observed. A significant sugar residue remained after fermentation, which aligns with findings from previous studies—reduction in sugar levels following the saccharification phase and 24 h of fermentation was minimal [6]. Ravyts et al. [54] observed similar results—after 24 h of rye sourdough fermentation, sucrose was no longer detected, while glucose, fructose, and maltose remained at high concentrations. In our study, a deficiency in fructose was confirmed when the glucose content remained unchanged. In contrast, the fructose content did not significantly decrease in rye scald fermented for 24 h at 30 °C with *Lactiplantibacillus paracasei* [7]. The decrease in sucrose indicates that sucrose is an essential substrate for LAB metabolism, which depends on strain, substrate availability, temperature, acidity, and time [15]. The high residual concentrations of maltose and glucose may be explained by the complexity of the substrate, the gradual solubility of compounds in wholemeal rye flour through enzymatic processes, and the potential amylolytic activity of LAB [7].

The sugar composition of bread produced using differently fermented exhibited variability in fructose, glucose, sucrose, maltose, and total sugar levels (Table 6).

The fructose content increased with extended fermentation times of the scald, starting at 0.37 ± 0.04 g 100 g^−1^ dw at RB0 and peaking at 0.89 ± 0.01 g 100 g^−1^ dw at RB24. Glucose content displayed negligible fluctuations, reaching its highest concentration at RB36 (0.56 ± 0.01 g 100 g^−1^ dw) and the lowest concentration at RB48 (0.43 ± 0.03 g 100 g^−1^ dw). Sucrose content demonstrated a consistent decrease with prolonged fermentation time, beginning at 0.50 ± 0.05 g 100 g^−1^ dw at RB0 and falling to the lowest amount of 0.30 ± 0.03 g 100 g^−1^ dw at RB48. The reduction in sucrose is also attributed to its hydrolysis into glucose and fructose, a process facilitated by yeast activity during dough fermentation [55]. Maltose, the predominant sugar in bread samples, showed a slight decreasing trend, starting at 13.32 ± 0.14 g 100 g^−1^ dw at RB0 and stabilizing around 12.11–12.12 g 100 g^−1^ dw from RB24 to RB48. The total sugar content remained relatively stable across all samples, with minor variations between.

The fermentation duration of scalds significantly influences the sugar profile in bread, particularly concerning fructose and sucrose, whereas maltose and glucose levels exhibit relative stability. The sugar content in bread was significantly lower compared to the scald. This indicates that sugars are abundantly produced during the saccharification phase of the scald, with only a small portion being consumed by lactic acid bacteria (LAB) or yeast during fermentation. Additionally, bread preparation involves adding extra flour, and the dough undergoes a shorter fermentation time than the scald.

#### 3.1.4. Effect of Fermentation Time on Phytic Acid Content in Rye Scald and Rye Bread

**Figure 5:**
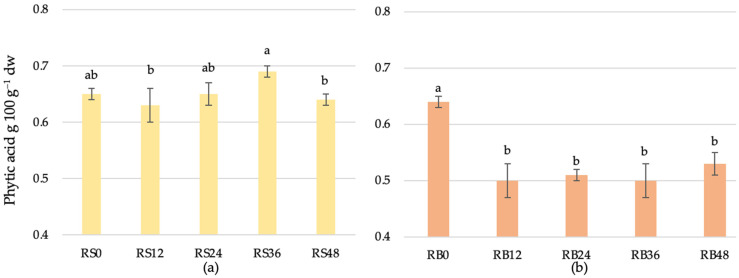
Phytic acid content of scald (**a**) fermented for different duration (RS0—unfermented, RS12—12 h, RS24—24 h, RS36—36 h, and RS48—48 h) and bread (**b**) (RB0—unfermented, RB12—12 h, RB24—24 h, RB36—36 h, and RB48—48 h fermented scald used). a–c indicate significant differences between the samples (*p* ≤ 0.05).

The initial phytic acid content in the scald was 0.65 ± 0.01 g 100 g^−1^ dry weight basis (dw) at 0 h (Figure 5). A slight reduction was observed at 12 h, with the content decreasing to 0.63 ± 0.03 g 100 g^−1^ dw. Interestingly, phytic acid levels were increased at 24 and 36 h, reaching 0.65 ± 0.02 g 100 g^−1^ and 0.69 ± 0.01 g 100 g^−1^, respectively. By 48 h, a marginal decrease was noted, with the phytic acid content measured at 0.64 ± 0.01 g 100 g^−1^. Haros et al. found that LAB from various sources optimally degrades phytic acid at a pH of 5–7 and temperatures of 50–70 °C [56]. However, no rapid phytic acid degradation was observed during scald fermentation, indicating the absence of phytase activity.

The initial phytic acid content in bread was recorded at 0.64 ± 0.01 g 100 g^−1^ dw with unfermented scald (Figure 5). A significant reduction occurred during the early stages of fermentation, with levels declining to 0.50 ± 0.03 g 100 g^−1^ (22%) by 12 h. Between 24 and 36 h, phytic acid content exhibited minimal variation. However, a slight increase was observed by 48 h, with the content rising to 0.53 ± 0.02 g 100 g^−1^.

Reale et al. [57] reported that the degradation of phytate is linked to endogenous phytase activity during the fermentation of wheat and rye dough. Lactic acid bacteria contribute to favorable conditions for phytase activity by reducing the pH. A moderate decrease in pH during sourdough fermentation creates an optimal environment for activating endogenous phytase enzymes present in the flour, which are primarily responsible for catalyzing phytate hydrolysis [58]. This acidification enhances enzymatic activity by aligning the pH with the optimal range for phytase function (pH around 5.5), thereby accelerating the breakdown of phytate and improving nutrient bioavailability in the sourdough matrix [27]. However, lactic acid bacteria possess specialized metabolism supported by an enzyme-producing system. Nuobariene et al. [26] highlighted both intracellular and extracellular enzymatic activity in various strains of *L.panis*, *L.reuteri*, and *L.fermentum* isolated from wheat and rye sourdough. Phytic-acid-rich substrates can enhance the metabolic activity of these bacteria through the action of intracellular phytase. Also, Cizeikiene et al. [10] found extracellular phytase activity in sourdough with thermophilic bacteria *L. bulgaricus*, *L. acidophylus*, and *L. rossiae* at pH < 3.5 (40 °C fermentation). In our study, a rapid decrease in phytic acid was not observed in the long-time fermentation of phytic acid with *Lactobacillus delbrueckii*. The results suggest that enzymatic degradation of phytic acid occurred in bread with fermented scald compared to bread with non-fermented scald, potentially due to optimal conditions (pH) for endogenous enzymes of flour, particularly the acidity of the fermented scald added to the dough and dough fermentation. The efficiency of phytate degradation is significantly influenced by fermentation conditions, particularly pH and temperature [59]. Yildirim et al. [60] found that the phytic acid content of wholegrain wheat bread fermented with sourdough decreased by 30.47– 69.76% depending on the dough fermentation temperature (25–35 °C), reaching the highest reduction at 25 °C. Haros et al. [56] determined that the optimal temperature range for phytase activity in various lactic acid bacteria, including *L. plantarum*, *L. fermentum*, and *L. casei*, is between 37 °C and 60 °C. Rodriguez et al. [61] investigated the influence of the bread-making process on phytase activity and found that sourdough bread exhibited a 25% greater reduction in myo-inositol hexakisphosphate content compared to bread made without sourdough or with sponge dough.

#### 3.1.5. Effect of Fermentation Time on Fructan Content in Rye Scald and Rye Bread

The analyzed rye flour was determined to have a fructan content of 3.31 ± 0.31 g 100 g^−1^ on a dry weight basis (dw). The fructan content in Finnish rye grains ranges from 4.6 to 6.6 g 100 g^−1^ dw, with commercial whole grain rye flour containing about 4 g 100 g of fructans [62]. In the study by Andersson et al. [63], the fructan content ranged from 3.6% to 4.6% of dry samples, with variations observed among the 18 analyzed cultivars of whole-grain rye.

**Figure 6:**
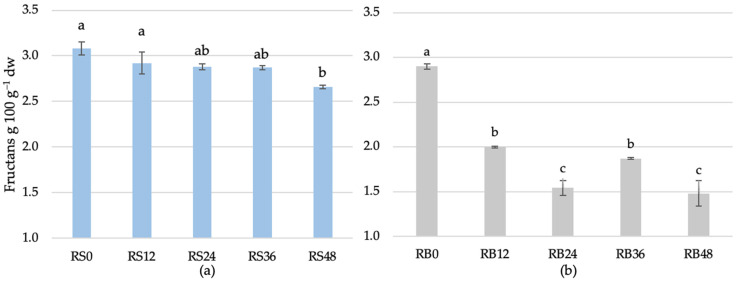
Fructan content of scald (**a**) fermented for different duration (RS0—unfermented, RS12—12 h, RS24—24 h, RS36—36 h, and RS48—48 h) and bread (**b**) (RB0—unfermented, RB12—12 h, RB24—24 h, RB36—36 h, and RB48—48 h fermented scald used). a–c indicate significant differences between the samples (*p* ≤ 0.05).

The variation in fructan content was analyzed in scalds subjected to fermentation for different durations and in bread produced from scalds fermented for varying time intervals. Initially, the fructan content in the fermented rye scald was 3.08 ± 0.07 g 100 g^−1^ dw at 0 h of fermentation (Figure 6). A progressive decline in fructan content was observed throughout the fermentation process. After 12 h, the fructan content had decreased to 2.92 ± 0.12 g 100 g^−1^ dw. This reduction continued steadily and was 2.66 g ± 0.02 100 g^−1^ dw at 48 h. While the reduction in fructan content was consistent throughout, the rate of decline appeared to diminish after 24 h, indicating a potential deceleration in fructan hydrolysis. During scald fermentation, fructans, a type of carbohydrate found in some plant sources, can undergo significant changes. The fermentation process typically involves the action of enzymes and microorganisms, which can lead to the breakdown of fructans into simpler sugars. The enzymes involved in the hydrolysis of fructans during fermentation include inulinase, fructosidase, levanase, and β-fructofuranosidase, depending on the specific strains of lactic acid bacteria used [64]. Lactic acid bacteria can use extracellular and intracellular pathways for the degradation of fructans [65,66]. The review article highlights that fructanases typically exhibit optimal activity at slightly acidic to neutral pH levels and within a temperature range 40–70 °C [35]. Fructan breakdown extracellularly by fructanases can result in the formation of fructose and enhance the overall sugar profile of the bread [32,67]. Studies have shown that sourdough fermentation with fructanase-positive lactic acid bacteria, like *Lactobacillus crispatus*, *Lactobacillus farciminia*, *Lactobacillus plantarum*, *Lactobacillus brevis*, and *Lactobacillus fermentum*, can significantly degrade fructans in bread [34,64]. Interestingly, according to Takagi et al.’s [68] study, *Lactobacillus delbrueckii* (source of yogurt) are able to grow in vitro inulin system and demonstrate their ability to internalize intact inulin and break down within their cells through intracellular processes. Pejcz et al. [18] found that the fructan content in rye sourdough significantly decreases depending on the LAB species used and fermentation time. After 48 h of fermentation, the content of fructans decreased from 3.7 to 1.2 g 100 g^−1^ dw with *Lactiplantibacillus plantarum* and to 1.06 g 100 g^−1^ dw with *Lacticaseibacillus casei*. In contrast, the rye bread sample prepared with 8% dry sourdough, intended as acidifier only to reduce the rye flours amylolytic activity, contained a significantly higher fructan content (2.3 g 100 g^−1^ dw).

The initial fructan content was slightly lower in bread prepared from unfermented rye scald—2.90 ± 0.03 g 100 g^−1^ dw. During fermentation, a significant reduction in fructan content was noted, with levels decreasing to 2.00 ± 0.01 g 100 g^−1^ dw after 12 h. Further reductions were observed at subsequent time points, with concentrations of 1.54 ± 0.08 g 100 g^−1^ dw at 24 h, 1.87 ± 0.01 g 100 g^−1^ dw at 36 h, and 1.48 ± 0.14 g 100 g^−1^ dw at 48 h.

Although the overall trend showed a progressive reduction in fructan content, fluctuations at 36 and 48 h suggest the potential impact of enzymatic or microbial processes. These changes can be attributed to shifts in microbial activity, changes in enzymatic efficiency, and alterations in the substrate, highlighting the complexity of fructan degradation in this system. The unfermented scald, which contained the highest fructan content, influenced the fructan levels in bread. Extended fermentation of the flour can effectively reduce the fructan content in the bread. Fructan degradation in bread occurs more quickly due to the sourdough microbiota used in dough fermentation. Research has indicated that invertase produced by the yeast *Saccharomyces cereviseae* facilitates partial fructan hydrolysis [69]. Extracellular fructanases are identified as the key enzymes involved in fructan degradation, with their production being species-dependent. *S. cerevisiae* does not express extracellular fructanase, whereas *K. marxianus* exhibits positive extracellular fructanase activity [30]. Therefore, the variations in fructan content observed during bread preparation are likely due to the combined enzymatic activities of both endogenous enzymes and those produced by the fermenting microbiota, leading to the hydrolysis of fructans into simpler sugars consumed during fermentation [70]. The fructan content in rye bread can be influenced by several factors, including the type of flour used, the fermentation process, and adding other ingredients.

### 3.2. Sensory Evaluation of Bread with Scald Fermented for Different Durations

**Figure 7:**
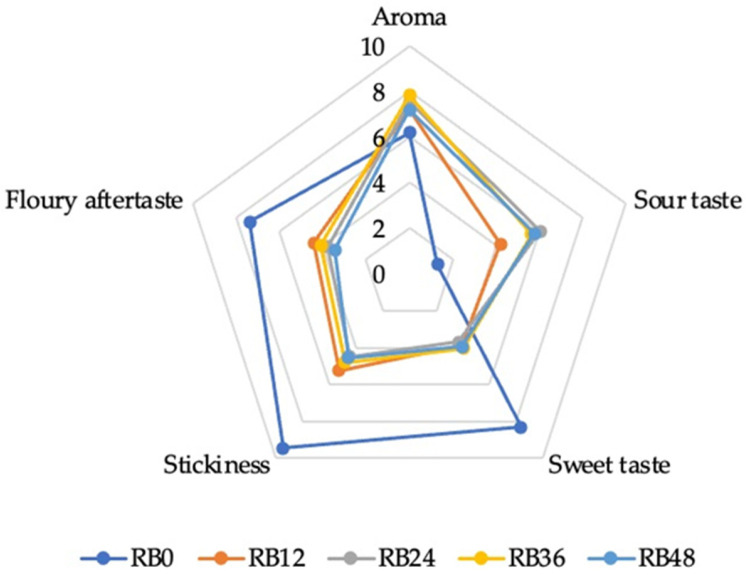
Mean sensory intensity scores for rye bread prepared with scalds fermented for different durations (RB0: unfermented, RB12: fermented for 12 h, RB24: 24 h, RB36: 36 h, and RB48: 48 h).

The sensory evaluation of bread samples (RB0, RB12, RB24, RB36, and RB48) revealed changes in sensory attribute intensity across aroma, sour taste, sweet taste, stickiness, and floury aftertaste. Compared to bread with unfermented scald, bread with fermented scald exhibited improved aroma, reduced sweetness, decreased stickiness, and produced a milder floury aftertaste. Furthermore, when the scald was fermented for 24 h or more, the sour taste of the bread samples became more balanced and stable (Figure 7).

For aroma, the highest scores were observed in samples RB36 (7.84 ± 1.95) and RB48 (7.08), indicating a more intense aroma in bread made with longer fermentation times compared to the unfermented bread (RB0, 5.53). The intensity of the sour taste increased with fermentation time. The lowest sour taste intensity was observed in RB0, with a significant increase in samples fermented for 12 h (RB12, 4.22 ± 2.01) and 24 h (RB24, 6.03 ± 1.65). No significant differences were observed in sour taste intensity among RB24, RB36, and RB48, suggesting that extending the fermentation time beyond 24 h does not substantially enhance sourness and that fermentation stabilizes after this duration. Sweetness was highest in the control sample (RB0, 8.31 ± 2.62) and decreased progressively with longer fermentation times and was 3.95 ± 2.25 using 48 h fermented scald (RB48). Stickiness was most pronounced in the unfermented bread (RB0, 9.44 ± 1.96) and reduced during fermentation. No significant differences existed between samples RB12, RB2, RB36, and RB48. Floury aftertaste showed that RB0 (7.38 ± 2.12b) was significantly distinct from the other samples, which all shared similar scores. This suggests that extended fermentation times (12–48 h) reduced the perception of floury aftertaste, enhancing the sensory acceptability of the bread.

The results demonstrate that fermentation time substantially influences the sensory properties of bread. RB0 was prepared with unfermented scald, meaning there was no acidity present. However, active enzymes from rye flour and malt were functional during the scalding process, resulting in sugar degradation, dextrin formation, and starch gelatinization during scalding. These enzymatic activities contributed to distinct sensory attributes in the RB0 bread sample. In contrast, the other samples underwent fermentation for varying durations (12–48 h), which introduced acidity, altered sugar composition, and modulated the sensory profile. This aligns with the absence of acidity in the unfermented bread and the rapid increase in acidity observed in the scald and bread after 12 h of fermentation. This can be explained by the LAB activity in the fermented scald. Scald fermentation with LAB positively affected sensory properties, as confirmed by Cizeikiene et al. [10], who demonstrated that LAB fermentation influenced the volatile composition of wholemeal wheat sourdough and bread. Bread made with unfermented scald (RB0) exhibited higher sweetness, stickiness, and a pronounced floury aftertaste, whereas prolonged fermentation (36–48 h) enhanced aroma and balanced sourness without significantly affecting sweetness or crumb stickiness. These findings underscore the potential for optimizing fermentation duration to achieve desired sensory properties in rye bread formulations.

## 4. Conclusions

The fermentation time of up to 48 h plays a crucial role in affecting the physicochemical properties of rye scald and the overall quality of the bread. A rapid decrease in pH observed after 12 h of fermentation and increased TTA indicated that scald is a suitable substrate for *L. delbrueckii* metabolism. The lactic acid content in scald increased significantly, from 0.27 ± 0.01 g 100 g^−1^ dw after 12 h to 3.72 ± 0.32 g 100 g^−1^ dw after 48 h, representing a 13.8-fold increase. The acidity generated during scald fermentation created favorable conditions for subsequent combined scald and dough fermentation, resulting in a 20% reduction in phytic acid, which enhances mineral bioavailability, and a 49% reduction in fructans, making the bread more suitable for individuals sensitive to FODMAPs.

Although fermentation time had no observable effect on specific parameters, it notably improved the bread’s sensory qualities. Extended fermentation reduced stickiness, balanced sweet and sour flavors, enhanced aroma, and minimized the floury aftertaste. The lack of significant differences observed when increasing scald fermentation time from 24 to 48 h provides an opportunity to optimize the production process while maintaining the desired quality of the bread.

Understanding these biochemical, nutritional, and sensory changes during fermentation can optimize the process, ensuring it improves the bread’s nutritional value and aligns with consumer preferences for flavor, texture, and aroma.

## Figures and Tables

**Table 1:** Parameters of sourdoughs for scald and dough fermentation.

Parameter	Sourdough Scald	Sourdough Dough
pH	3.5 ± 0.0	3.7 ± 0.0
TTA, mL 0.1 N NaOH	16.7 ± 0.2	17.7 ± 0.6
LAB, log_10_ CFU g^−1^	8.9 ± 0.1	8.6 ± 0.1
Yeasts, log_10_ CFU g^−1^	-	7.1 ± 0.3

Sourdough Scald—sourdough for scald fermentation; Sourdough Dough—sourdough for dough fermentation; TTA—total titratable acidity; CFU—colony forming units.

**Table 2:** Recipe for scald and bread preparation.

Ingredients, kg	Scald	Dough
Rye wholemeal flour	6.00	8.00
Rye diastatic malt	0.06	-
Water (92 °C)	9.00	-
Sourdough Scald	0.12	-
Sourdough Dough	-	0.40
Salt	-	0.11
Scald	-	15.18
Total	15.18	23.69

**Table 3:** Profile of organic acid in 48 h scald fermentation.

Organic Acids, g 100 g^−1^ dw	Scald Samples				
	RS0	RS12	RS24	RS36	RS48
Lactic acid	0.27 ± 0.01 c	2.83 ± 0.07 b	3.74 ± 0.16 a	3.85 ± 0.03 a	3.72 ± 0.32 a
Acetic acid	0.03 ± 0.01 b	0.12 ± 0.02 a	0.01 ± 0.00 b	0.01 ± 0.00 b	0.01 ± 0.00 b
Propionic acid	ND	ND	0.01 ± 0.00 a	0.01 ± 0.00 a	0.01 ± 0.00 a
Butyric acid	0.09 ± 0.02 b	0.18 ± 0.01 a	0.01 ± 0.00 c	0.01 ± 0.00 c	0.01 ± 0.00 c
Succinic acid	0.07 ± 0.01 b	0.16 ± 0.01 a	0.14 ± 0.00 a	0.14 ± 0.02 a	0.13 ± 0.01 a
Oxalic acid	0.03 ± 0.00 a	0.03 ± 0.00 a	0.03 ± 0.01 a	0.03 ± 0.01 a	0.04 ± 0.02 a
Quinic acid	0.55 ± 0.03 a	0.76 ± 0.07 a	0.72 ± 0.16 a	0.78 ± 0.13 a	0.74 ± 0.21 a
Tartaric acid	0.21 ± 0.02 a	0.25 ± 0.03 a	0.24 ± 0.06 a	0.28 ± 0.06 a	0.25 ± 0.06 a
Malic acid	0.20 ± 0.01 a	0.12 ± 0.01 b	0.11 ± 0.00 b	0.10 ± 0.01 b	0.11 ± 0.01 b
TOTAL	1.45 ± 0.04 b	4.46 ± 0.16 a	5.01 ± 0.40 a	5.22 ± 0.20 a	5.02 ± 0.02 a

dw—dry weight; ND—not detected; rye scald fermented for varying durations: RS0—unfermented, RS12—12 h, RS24—24 h, RS36—36 h, and RS48—48 h. a–c indicate significant differences between the samples (*p* ≤ 0.05) in a row.

**Table 4:** Profile of organic acids in rye bread prepared with scald fermented for varying durations.

Organic Acids, g 100 g^−1^ dw	Bread Samples				
	RB0	RB12	RB24	RB36	RB48
Lactic acid	0.81 ± 0.0 6 c	1.54 ± 0.04 b	1.91 ± 0.13 ab	2.06 ± 0.12 a	2.00 ± 0.13 ab
Acetic acid	0.23 ± 0.03 a	0.17 ± 0.03 a	0.19 ± 0.01 a	0.16 ± 0.02 a	0.21 ± 0.03 a
Propionic acid	0.08 ± 0.02 a	0.01 ± 0.00 a	0.07 ± 0.03 a	0.06 ± 0.02 a	0.04 ± 0.03 a
Butyric acid	0.41 ± 0.05 a	0.29 ± 0.01 a	0.36 ± 0.03 a	0.30 ± 0.01 a	0.38 ± 0.03 a
Succinic acid	0.03 ± 0.01 b	0.13 ± 0.01 a	0.16 ± 0.00 a	0.15 ± 0.00 a	0.17 ± 0.03 a
Oxalic acid	0.03 ± 0.00 a	0.03 ± 0.01 a	0.03 ± 0.01 a	0.03 ± 0.01 a	0.03 ± 0.01 a
Quinic acid	0.84 ± 0.08 a	0.77 ± 0.23 a	0.82 ± 0.16 a	0.86 ± 0.11 a	0.84 ± 0.17 a
Tartaric acid	0.24 ± 0.05 a	0.19 ± 0.05 a	0.20 ± 0.04 a	0.20 ± 0.01 a	0.21 ± 0.03 a
Malic acid	0.27 ± 0.02 b	0.30 ± 0.03 ab	0.34 ± 0.03 ab	0.36 ± 0.02 a	0.35 ± 0.01 ab
TOTAL	2.94 ± 0.10 b	3.43 ± 0.33 ab	4.08 ± 0.31 ab	4.17 ± 0.20 a	4.23 ± 0.40 a

dw—dry weight; ND—not detected; rye bread prepared with scald fermented for varying durations: RB0—unfermented, RB12—12 h, RB24—24 h, RB36—36 h, and RB48—48 h fermented scald used. a–c indicate significant differences between the samples (*p* ≤ 0.05) in a row.

**Table 5:** Profile of sugars (g 100 g^−1^ dw) in rye scald prepared for varying durations.

Sugars, g 100 g^−1^ dw	Scald Samples				
	RS0	RS12	RS24	RS36	RS48
Fructose	0.40 ± 0.01	ND	ND	ND	ND
Glucose	0.99 ± 0.05 ab	0.72 ± 0.08 b	0.87 ± 0.15 ab	0.88 ± 0.01 ab	1.08 ± 0.01 a
Sucrose	1.01 ± 0.09 a	0.50 ± 0.03 b	0.31 ± 0.04 bc	0.36 ± 0.02 bc	0.25 ± 0.01 c
Maltose	25.33 ± 0.62 b	27.56 ± 0.12 a	26.6 ± 0.15 ab	27.31 ± 1.06 ab	26.99 ± 0.03 ab
Unknown	3.05 ± 0.13 b	4.47 ± 0.40 a	4.86 ± 0.21 a	4.89 ± 0.32 a	4.21 ± 0.34 a
TOTAL	30.78 ± 0.09 b	33.25 ± 0.63 a	32.64 ± 0.54 ab	33.44 ± 1.42 ab	33.53 ± 0.39 ab

dw—dry weight; ND—not detected; rye scald fermented for varying durations: RS0—unfermented, RS12—12 h, RS24—24 h, RS36—36 h, and RS48—48 h. a–c indicate significant differences between the samples (*p* ≤ 0.05) in a row.

**Table 6:** Profile of sugars (g 100 g^−^^1^ dw) in rye bread prepared with scald fermented for varying durations.

Sugars, g 100 g^−1^ dw			Bread Samples		
	RB0	RB12	RB24	RB36	RB48
Fructose	0.37 ± 0.04 b	0.63 ± 0.01 a	0.89 ± 0.01 a	0.57 ± 0.02 a	0.67 ± 0.01 a
Glucose	0.49 ± 0.06 ab	0.45 ± 0.03 a	0.46 ± 0.01 ab	0.56 ± 0.01 ab	0.43 ± 0.03 b
Sucrose	0.50 ± 0.05 a	0.32 ± 0.06 b	0.31 ± 0.05 bc	0.37 ± 0.06 bc	0.30 ± 0.03 c
Maltose	13.32 ± 0.14 a	12.62 ± 0.15 b	12.20 ± 0.18 ab	12.11 ± 0.12 ab	12.12 ± 0.27 ab
Unknown	1.33 ± 0.01 b	2.13 ± 0.10 a	2.03 ± 0.22 a	2.27 ± 0.05 a	2.23 ± 0.10 a
TOTAL	16.58 ± 0.42 a	16.82 ± 0.37 a	16.56 ± 0.47 a	16.54 ± 0.26 a	16.41 ± 0.45 a

dw—dry weight; rye bread prepared with scald fermented for varying durations: RB0—unfermented, RB12—12 h, RB24—24 h, RB36—36 h, and RB48—48 h fermented scald used. a–c indicate significant differences between the samples (*p* ≤ 0.05) in a row.

## Data Availability

The original contributions presented in the study are included in the article; further inquiries can be directed to the corresponding author.
